# Reduction of Blood Transfusion in Iron Deficiency Anemia (ReBIDA): A Quality Improvement Initiative

**DOI:** 10.1155/ah/5513287

**Published:** 2025-06-17

**Authors:** Andrew Picca, David Kling, Amanda Jacobson-Kelly, Kathleen Nicol, Joseph Stanek, Vilmarie Rodriguez

**Affiliations:** ^1^Division of Pediatric Hematology/Oncology/BMT, The Ohio State University College of Medicine, Nationwide Children's Hospital, Columbus, Ohio, USA; ^2^Division of Emergency Medicine, The Ohio State University College of Medicine, Nationwide Children's Hospital, Columbus, Ohio, USA; ^3^Divison of Pathology and Laboratory Medicine, The Ohio State University College of Medicine, Nationwide Children's Hospital, Columbus, Ohio, USA

## Abstract

**Background:** Iron deficiency anemia (IDA) is the most common form of pediatric anemia, with first-line treatment focusing on iron repletion through oral and/or intravenous iron. The American Society of Hematology (ASH)/the American Society of Pediatric Hematology and Oncology (ASPHO) Choosing Wisely Campaign recommends against packed red blood cell (PRBC) transfusion for asymptomatic IDA. PRBCs are a finite resource and carry treatment associated risk compared to iron therapies. The use of oral and intravenous iron is an effective, tolerated therapy modality for IDA which can be overlooked based on the degree of anemia.

**Study Design and Methods:** Plan, Do, Study, Act methodology was used for this single institution quality improvement initiative. The objective was to decrease the percentage of PRBC transfusions in all admitted IDA patients from a baseline of 72% to a target of 50% by December 2022 and to sustain for 12 months. Interventions consisted of multidisciplinary, evidence-based didactic education sessions and development of a single institution clinical practice guideline for the treatment of IDA.

**Results:** In the pre-education/baseline group, 72% (*n* = 57/79) of patients received PRBC transfusion for the treatment of IDA, compared to the posteducation/intervention group where 38% (*n* = 29/76) of patients received PRBC transfusion for the treatment of IDA (*p* value < 0.0001). In the pre-education/baseline group, 19% (*n* = 11/57) of patients received PRBC transfusions not indicated based on the developed CPG, compared to 6.9% (*n* = 2/18) in the posteducation/intervention group (*p* value = 0.20).

**Discussion:** This work demonstrates how multidisciplinary, education- and evidence-based interventions lead to clinically and statistically significant reductions in PRBC transfusion for admitted patients with IDA.

## 1. Introduction

Iron deficiency anemia (IDA) is the most common form of pediatric anemia worldwide [1]. Severe IDA may lead to cognitive defects, cerebral sinus venous thrombosis with or without cerebrovascular hemorrhage, or high-output cardiac failure [2]. Risk factors for IDA include excessive cow's milk intake, poor dietary iron, menstrual blood loss, other chronic blood losses (e.g., inflammatory bowel disease), and intestinal malabsorption disorders [1, 2]. Diagnosing IDA relies on history, physical, and laboratory evaluation. Patients can be asymptomatic without frank symptoms due to slow clinical progression of IDA and physiologic compensation for the degree of anemia [3, 4].

Replacement of iron stores and addressing underlying pathophysiologic mechanisms of iron deficiency are essential for the treatment of IDA [5]. Oral iron replacement with 3 mg/kg/day of elemental iron in one daily dose is the first-line treatment [5, 6]. Every other day, oral iron dosing is an acceptable regimen [4]. There are multiple formulations of intravenous (IV) iron which have Food and Drug Administration approval for children with IDA [5]. IV iron is a safe alternative for children unsuccessfully treated with oral iron, or having contraindications to its use [7]. Side effects from IV iron tend to be transient, with a low risk of serious reactions [5, 7, 8].

Packed red blood cell (PRBC) transfusion can be used to treat patients with IDA having hemodynamic instability and active bleeding [4, 6]. PRBC transfusion does not adequately treat iron deficiency, as iron transfused with PRBCs is not readily bioavailable. Acute adverse events from PRBC transfusions include risks of bloodborne viral infection transmission, transfusion reactions, and cardiac volume overload, while long-term adverse events include alloantibody formation [4, 9]. The American Society of Hematology (ASH) and the American Society of Pediatric Hematology and Oncology (ASPHO) recommend against the use of PRBCs for the treatment of IDA in patients without hemodynamic instability or active bleeding [4, 6]. PRBCs are a finite resource, demonstrating the importance of adherence to appropriate practice guidelines to limit overuse.

IDA is a disease process which spans not only pediatric hematology but other pediatric care subspecialties, marking the importance in educating colleagues outside of pediatric hematology. Through quality improvement (QI) initiatives, our primary objective was to decrease the percentage of PRBC transfusion, at Nationwide Children's Hospital, in all hospital admitted patients with IDA from a baseline of 72% transfusion to 50% transfusion by December 2022 and to sustain for 12 months. Secondary outcomes compared transfusion rates before and after our QI interventions to better direct iterative interventions. The methodology, results, and discussion described here were presented at the ASPHO 2023 Spring Conference as an oral abstract presentation and published in partial as an abstract in the 2023 ASPHO Conference Paper and Poster Index supplementary issue of *Pediatric Blood and Cancer* [10].

## 2. Methods

**Figure 1:**
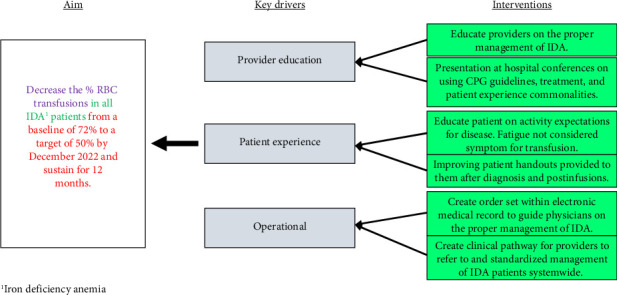
Key driver diagram.

Plan, Do, Study, Act (PDSA) methodology was used for this QI initiative. This research was exempted from the Institutional Review Board approval, given its quality improvement nature. The population of interest was hospital-admitted patients with the primary diagnosis of IDA. A list of all potential stakeholders was outlined by the pediatric hematology authors. Identified key stakeholders within pediatric hematology, pediatric emergency medicine, and pediatric pathology (transfusion medicine) were invited to participate in this quality improvement initiative. These specialties held impact potential to limiting the use of PRBCs in IDA treatment. These groups were motivated to improve patient care, provide fiscally responsible care, and improve system efficiency. Stakeholders identified key drivers through the current care practices and workplace experiences and developed interventions focusing on key drivers to achieve the primary objective (Figure 1).

Patients admitted with the primary diagnosis of IDA were identified retrospectively using ICD10 codes for hospital admission on the hematology, gastrointestinal, surgical, medical surgical, and intensive care units. Inpatient units did not individually reach out to the study team regarding admission of patients meeting criteria for inclusion. Clinical data were collected from March 2019 through February 2023. Data included baseline demographic data, hemoglobin on admission, interventions (e.g., blood transfusion, oral iron therapy, and IV iron therapy), and underlying etiology of IDA. QI interventions were implemented in March 2022. The preintervention or baseline data were defined as patients with the primary diagnosis of IDA admitted from March 2019 through February 2022, and the postintervention group defined as patients with the primary diagnosis of IDA admitted from March 2022 through February 2023. The identified chronological delineations of pre- and postintervention groups were based on accrual hospital admissions with the primary diagnosis of IDA in coordination with timeline for planned educational interventions. Longitudinal data were followed using a p-chart to illustrate the proportion of patients with the diagnosis of IDA being transfused with PRBCs. Statistical comparisons between the pre- and postintervention groups were made using chi-squared tests with *p* values < 0.05 considered significant.

The QI interventions implemented in March 2022 focused on education across multidisciplinary teams. Stakeholders prioritized education across multidisciplinary teams as a QI intervention to promote provider autonomy and decision-making outside of their specialty, further promoting self-directed education to other colleagues in similar fields. Stakeholders felt long-lasting improvements driven by education would be superior to other methodologies given time constraints of those receiving education. Structured, evidence-based didactic education was provided to the adolescent medicine, emergency medicine, transfusion medicine, gastroenterology, hematology, and pediatric hospital medicine services. Education focused on pathophysiology, laboratory evaluation for IDA, and review of available treatment options for IDA at Nationwide Children's Hospital.

A clinical practice guideline (CPG) was drafted with the goal to provide structured, evidence-based treatment recommendations for symptomatic and asymptomatic patients with IDA. These recommendations were shared during didactic education. Subspecialty feedback on the CPG was incorporated into subsequent iterations of the internal CPG drafts after stakeholder discourse within the framework of evidence-based support. The CPG for management of IDA was locally published in October 2022 and focused on diagnosing IDA, laboratory, and clinical parameters where treatment with PRBC was indicated, and guidance on oral and IV iron dosing and administration. The internally published CPG was revised, addended, and updated prior to creating the publicly available IDA clinical pathway which was published following this work.

## 3. Results

Demographic data of the pre- and postintervention cohorts are presented in Table 1. Demographic data are also compared between patients who received and did not receive PRBC transfusion in both groups. There were statistically significant differences in admission hemoglobin between the no transfusion and transfusion groups in both the pre- and postintervention groups. Patients in the study were admitted through the Nationwide Children's Hospital emergency department and presented as referrals from primary care and subspecialty providers in and out of the Nationwide Children's network, or for patient symptoms requiring emergent medical care.

**Figure 2:**
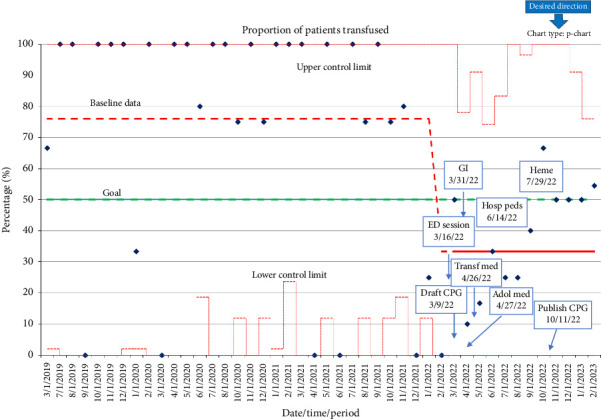
Proportion of patients transfused [10].

Preintervention data demonstrated a transfusion rate of 72%. Following QI interventions, monthly transfusion rate data were collected through February 2023 and analyzed with use of a p-chart (Figure 2). Six consecutive points on the same side of the center line (average) are required to lead to a process shift, delineating a new running average. The ongoing average of transfusion following QI interventions demonstrated a process shift to a mean of 32% transfusion rate at the time of March 2022.

The preintervention group had a 72.2% transfusion rate (57/79). Of the transfused group, patients were admitted for IDA secondary to blood loss related to menses (45.6%), IDA due to limited dietary iron (22.8%), gastrointestinal bleeding (19.3%), other bleeding symptoms (3.5%), or other etiologies (8.8%). Other etiologies included patients with short gut syndrome, gastric bypass surgery, and active concurrent infections with IDA. There was statistical significance of etiology for admission between nontransfused and transfused groups (*p* value 0.044). There was no statistical difference among admitting hospital services between the nontransfused and transfused groups, with most transfused patients admitted to the hematology (25/57) and adolescent medicine services (10/57). There was no statistically significant difference in those who received IV and/or oral iron between the transfused and nontransfused groups (Table 1).

The postintervention group had a 38.2% transfusion rate (29/76). Of the transfused group, patients were admitted for IDA secondary to blood loss related to menses (51.7%), IDA due to limited dietary iron (6.9%), gastrointestinal bleeding (27.6%), and other etiologies (13.8%). Other etiologies included patients with chronic renal disease, incidental findings of IDA, and active concurrent infections with IDA. There was no statistical significance of etiology for admission between nontransfused and transfused groups (*p* value 0.06). There was statistical significance seen among admitting services between the nontransfused and transfused groups (*p* value 0.0008), with most patients admitted to hematology (27.6%), adolescent medicine (34.5%), and gastroenterology (24.1%). There was no statistically significant difference in those who received IV and/or oral iron between the nontransfused and transfused groups (Table 1).

The pre- and postintervention groups were retrospectively screened against the developed CPG. The CPG recommended PRBC transfusion in 49/79 (60.7%) of preintervention patients, and 32/76 (42.1%) of postintervention patients. In direct comparison, there was a statistically significant reduction in the transfusion rate between the preintervention (72.1%) and the postintervention (38.2%) groups (*p* value < 0.0001). Of those transfused, 11 (19.2%) in the preintervention group and two (6.9%) in the postintervention group received PRBCs not indicated by the CPG (*p* value 0.20). In these instances, patients were transfused based on the laboratory value of hemoglobin or symptoms which were not consistent with the proposed definition of symptomatic anemia. There was statistically significant reduction in the percentage of patients transfused for the diagnosis of IDA due to limited dietary iron (62%–13%, *p* value 0.0071) and gastrointestinal bleeding (92%–40%, *p* value 0.0079) between the pre- and postintervention groups.

## 4. Discussion

IDA spans all general and subspecialty pediatric populations. This work demonstrates that the use of multidisciplinary, education- and evidence-based interventions leads to clinically and statistically significant reductions in PRBC transfusion for admitted patients with IDA. In this cohort, the subpopulations of children with IDA due to limited dietary iron intake and gastrointestinal bleeding benefitted most from interventions. Adolescents experiencing abnormal uterine bleeding or blood loss related to menses saw less reduction in PRBC transfusion, likely secondary to symptoms of active bleeding necessitating PRBC transfusion.

In promoting clinical practice changes among different subspecialties, barriers were overcome in a collaborative and multidisciplinary manner. The administration of IV iron in the emergency department was achieved through extensive education, discourse, and problem solving with the physician and nursing leadership in the emergency department. Open dialog and flexibility by the stakeholders were necessary to provide worthwhile education, while also being respectful of colleague's time constraints in an academic medical center.

This study has several limitations. There were significant differences in the duration of time required for similar cohort sizes in the pre- and postintervention groups to be obtained. The COVID-19 pandemic limited access to health care and routine screening visits during the primary intervention period of this work. It is postulated that the COVID-19 pandemic contributed to the discrepancy in time to accrue similar sized preintervention and postintervention cohorts, accrued over 3 years and approximately 1 year, respectively. Our changes and data points below the mean process shift were not sustained, likely secondary to the medical need for some patients to receive PRBCs in the setting of IDA. This could have been better addressed through a different primary aim. The use of pre- and postintervention group analysis was used to control this variable.

Five patients in the postintervention cohort where blood transfusion was indicated did not receive PRBC transfusion and were treated with IV and oral iron therapy. We acknowledge continued need for educational interventions. The initial, internal CPG is now a published hospital wide, publicly available clinical pathway as part of our ongoing initiative for providing evidence-based materials to guide IDA management (supporting information [available here]). The emergency department IDA clinical pathway has different inclusion and exclusion criteria as compared to the internal CPG, explaining the discrepancy in populations included in this study versus those included in the attached clinical pathway.

Limiting PRBC transfusion reduces transfusion-associated risks. Risks of transfusion to all populations include transfusion reactions, unnecessary financial burden to the patient and medical institution, and improper use of a strained medical resource. Adolescent females were the majority of admitted patients. It is prudent to limit unnecessary transfusion in this subpopulation, as female patients demonstrate higher likelihoods of alloantibody formation compared to males [11]. Women with alloantibody production who become pregnant are at higher risk of having children with hemolytic disease of the newborn [12].

By implementing education- and evidence-based approaches to PRBC transfusions in patients with IDA through quality improvement, we have decreased PRBC transfusions. The average cost for one unit of PRBCs is approximately $600, with cost of transfusion approximately $2000 per unit [13]. The postintervention group received 28 fewer transfusions, leading to an estimated, average healthcare savings of $72,800 ($2600 per transfusion) over 12 months [13]. Our work significantly decreased healthcare cost and preserved PRBCs for patients with life-threatening conditions.

The updated, secondary aim is to decrease the percentage of inappropriate PRBC transfusions as defined by our CPG from the new baseline of 6.9% identified in the postintervention group to 0% by July 2023 and sustain for 12 months. Future interventions will focus on service-directed education based on transfusion rates, creation of IDA diagnosis and management simulation center educational resource for the pediatric and emergency department residency programs, and engage local primary care providers with education on diagnosis and management of IDA. Expanding our aims to include decreasing the number of inappropriately withheld transfusions as defined by our CPG, from a baseline of five occurrences to zero, is a targeted area of data collection and evaluation in future PDSA cycles.

Treatment with oral and IV iron resolves underlying iron deficiency and corrects anemia, while PRBC transfusion is reserved for patients with active bleeding and hemodynamic instability [4–7]. Inappropriate use of PRBCs exposes patients to unnecessary risk and cost. In conclusion, implementation of evidence-based clinical guidelines and multidisciplinary education provides framework to minimize PRBC transfusions for asymptomatic patients with IDA.

## Figures and Tables

**Table 1:** Pre- and postintervention demographic comparison between transfusion and no transfusion [10].

**Preintervention demographics**	**No transfusion** **N = 22**	**Transfusion** **N = 57**	**p** **v**alue^**d**^

Age, years median (IQR)	11.5 (2–15)	13 (7–16)	0.18
Female sex *n* (%)	14 (63.6)	46 (80.7)	0.11
Admission hemoglobin (g/dL) median (IQR)	6.6 (4.9–8.4)	5.4 (3.8–6.5)	0.0080
Diagnosis group *n* (%)			0.0444
Blood loss related to menses	6 (27.3)	26 (45.6)	
Iron deficiency related to diet	8 (36.4)	13 (22.8)	
GI bleeding	1 (4.5)	11 (19.3)	
Other bleeding^a^	4 (18.2)	2 (3.5)	
Others^b^	3 (13.6)	5 (8.8)	
Admitting service *n* (%)			0.11
Pediatric hematology	15 (68.2)	25 (43.9)	
Pediatric hospital medicine	5 (22.7)	7 (12.3)	
Adolescent medicine	1 (4.5)	10 (17.5)	
Pediatric intensive care	0 (−)	7 (12.3)	
Pediatric gastroenterology	0 (−)	5 (8.8)	
Pediatric infectious disease	1 (4.5)	2 (3.5)	
Pediatric surgery	0 (−)	1 (1.8)	
IV iron *n* (%)	16 (72.7)	29 (50.9)	0.08
Oral iron on discharge *n* (%)	10 (45.5)	37 (64.9)	0.11

**Postintervention demographics**	**No transfusion** **N = 47**	**Transfusion** **N = 29**	**p value**

Age, years median (IQR)	13 (3–16)	14 (11–16)	0.57
Female sex *n* (%)	31 (66.0)	21 (72.4)	0.56
Admission hemoglobin (g/dL) median (IQR)	8.4 (6.6–9.5)	5.4 (4.7–6.4)	< 0.0001
Diagnosis group *n* (%)			0.06
Blood loss related to menses	12 (25.5)	15 (51.7)	
Iron deficiency related to diet	14 (29.8)	2 (6.9)	
GI bleeding	12 (25.5)	8 (27.6)	
Incidental finding related to cardiorespiratory symptoms	3 (6.4)	2 (6.9)	
Others^c^	6 (12.8)	2 (6.9)	
Admitting service *n* (%)			0.0008
Pediatric hematology	13 (27.7)	8 (27.6)	
Pediatric hospital medicine	17 (36.2)	1 (3.4)	
Adolescent medicine	3 (6.4)	10 (34.5)	
Pediatric intensive care	1 (2.1)	2 (6.9)	
Pediatric gastroenterology	12 (25.5)	7 (24.1)	
Pediatric nephrology	1 (2.1)	1 (3.4)	
IV iron *n* (%)	35 (74.5)	23 (79.3)	0.63
Oral iron on discharge *n* (%)	25 (53.2)	11 (37.9)	0.20

^a^Other bleeding includes acute ITP, AVM/penile bleeding, and thrombocytopenia.

^b^Others include short gut syndrome, gastric sleeve, DVT, active TB infection, chronic osteomyelitis, appendicitis, and unknown etiology.

^c^Others include pulmonary embolism, incidental finding, unknown etiology, focal segmental glomerular sclerosis, chronic IDA with renal disease, and sepsis.

^d^ *p* values result from Chi-square or Fisher's exact tests for categorical variables and Wilcoxon rank-sum for quantitative variables.

## Data Availability

The data that support the findings of this study are available from the corresponding author upon reasonable request.

## Supporting Information

The attached supporting file is the publicly published Nationwide Children's Hospital Iron Deficiency Anemia (IDA) Clinical Pathway. The Clinical Pathway provides providers with appropriate diagnostic assistance, management strategies, and disposition planning for patients meeting specific inclusion criteria with the diagnosis of IDA. This Clinical Pathway provides general guidance to clinicians; clinical judgment and other relevant factors in diagnosing and treating patients remain central to the selection of diagnostic tests and therapy.
