# Supplementary material for: Reduction of Blood Transfusion in Iron Deficiency Anemia (ReBIDA): A Quality Improvement Initiative
Source: Adv Hematol. 2025 Jun 17;2025:5513287. doi: 10.1155/ah/5513287 (PMC12187434; doi:10.1155/ah/5513287)
Supplement: Supporting Information — Additional supporting information can be found online in the Supporting Information section. [file 5513287.f1.pdf]

NATIONWIDE CHILDREN'S
When your child needs a hospital, everything matters.

Iron Deficiency Anemia
Emergency Department

Center for Clinical Excellence

Inclusion Criteria

≥ 6 months of age with history, symptoms or laboratory findings concerning for iron deficiency anemia (IDA)

Exclusion Criteria

- Active vaginal bleeding
- Traumatic etiology for blood loss
- Anemia related to chronic disease (e.g. oncologic, inflammatory, gastrointestinal)
- Anemia related to abnormal uterine bleeding

[Definitions]

[Risk Factors]

[Signs & Symptoms]

Concern for iron deficiency anemia based on history, physical exam, or prior labs

↓

If not already obtained, collect confirmatory testing

- Consider IV placement
  Place away from flexible joints if possible
- Type and screen if hypotensive or ill appearing
- CBC w/ Diff*
- Reticulocytes
- Phosphorous
- Iron w/ Total Iron Binding Capacity (TIBC)**
- Ferritin**

* Repeat if > 24 hours since last value
** Results might not be back prior to disposition

↓

Lab results concerning for IDA?

- No → **Off Pathway** — Consider Differential Diagnosis
- Yes ↓

Symptomatic anemia or **Hemoglobin (hgb) ≤ 4?**

- Yes → **Consult Hematology** ↓
  - **Any of the following?**
    - **Hgb < 3**
    - **Hypotension**
  - Yes → Initiate transfusion of RBCs 5 mL/kg (not to exceed 1 unit) *Run slowly over 3 hours* → **Admit to PICU**
  - No → **Admit to Hematology**
- No → **Treat based on Hgb level** ↓
  - **HGB < 6.1?**
  - Yes → **Consult Hematology** → Administer IV iron → **Discharge Home**
    - IV Iron Discharge Instructions
    - Oral iron supplementation
    - Follow up in 3-5 days with hematology
  - No → **Discharge Home**
    - Oral iron supplementation
    - Follow up in 1 week with primary care provider

Definitions

Anemia: Hemoglobin value below the 2.5% for age and biological gender, as per institutionally established laboratory standards (See table below – the lower end of the range represents the 2.5%)

Symptomatic Anemia: presence of one or more of the following symptoms with laboratory confirmed anemia:

- Shortness of breath
- Hypoxemia
- Acute respiratory failure
- Hypotension
- Syncope
- Positive orthostatic vital signs
- Sustained tachycardia
- To meet the definition of symptomatic, tachycardia should be observed in a calm and resting child.
- Age-appropriate values for heart rate should be used in assessment.

Asymptomatic Anemia: No evidence of any of the above noted symptoms

Test Name	Units	Age Range	Normal Range Male	Normal Range Female
Hemoglobin	g/dL	6M to <2Y	10.5-13.5	10.5-13.5
Hemoglobin	g/dL	2Y to <6Y	11.5-13.5	11.5-13.5
Hemoglobin	g/dL	6Y to <12Y	11.5-15.5	11.5-15.5
Hemoglobin	g/dL	12Y to <15Y	12.5-16.4	12.0-16.0
Hemoglobin	g/dL	15Y to <18Y	13.1-16.9	12.0-16.0

Algorithm

Risk Factors

- Excessive ingestion of cow's milk (> 24 oz of milk daily)
- Obesity
- Restrictive diets
- Menorrhagia
- Inflammatory Bowel Disease
- Severe and frequent epistaxis
- Gastrointestinal bleeding

Algorithm

Signs & Symptoms of Anemia

- Tachycardia
- Pallor (evaluate conjunctiva, oral mucosa and nail beds)
- Lethargy
- Poor feeding
- Cardiomegaly
- Tachypnea
- Pica

[Algorithm](#)

Differential Diagnosis

- Acute blood loss anemia
- Hemolytic anemia
- Thalassemia
- Viral suppression
- Leukemia
- Lead poisoning
- Renal Disease
- Hypothyroidism

Algorithm

Laboratory Testing

- All patients with concern for underlying iron deficiency anemia should have:
  - CBC with differential
  - Reticulocyte count
  - Iron with Total Iron Binding Capacity (TIBC)
  - Ferritin
  - Phosphorous level -- helpful in determining the optimal type of iron infusion
- Soluble transferrin receptor can aid in delineating iron deficiency from anemia of chronic disease/inflammation
- If concerned that a patient may require a PRBC transfusion, obtain type and screen

Algorithm

Laboratory Results Indicative of IDA

- Hemoglobin (HGB) < 2.5% for age and gender (lower number in range on table below)
- MCV < 80
- Low:
  - Iron
  - Transferrin Saturation
  - Ferritin
- High
  - Total Binding Iron Capacity (TIBC)
  - RDW

Test Name	Units	Age Range	Normal Range Male	Normal Range Female
Hemoglobin	g/dL	6M to <2Y	10.5-13.5	10.5-13.5
Hemoglobin	g/dL	2Y to <6Y	11.5-13.5	11.5-13.5
Hemoglobin	g/dL	6Y to <12Y	11.5-15.5	11.5-15.5
Hemoglobin	g/dL	12Y to <15Y	12.5-16.4	12.0-16.0
Hemoglobin	g/dL	15Y to <18Y	13.1-16.9	12.0-16.0

Algorithm

Iron Supplementation

Oral Iron Supplementation:

- If patient can swallow pills and is ≥ 35 kg:
  - Ferrous sulfate 325 mg (65 mg elemental iron) once per day x 3 months

- If patient cannot swallow pills or are < 35 kg:
  - Fer-in-sol 3 mg/kg of elemental iron once per day x 3 months

or

  - Novaferrum liquid (polysaccharide-iron complex) 3 mg/kg (max 325 mg) once per day x 3 months

Algorithm

Symptomatic Anemia

- Shortness of breath
- Sustained tachycardia
- Syncope
- Orthostatic hypotension *(fall of systolic BP over 20 mm Hg or fall in diastolic BP over 10 mmHg within 3 minutes of standing)*

Algorithm

Infusion Practice for Ferric Carboxymaltose (FCM)

- Indications for Ferric Carboxymaltose (FCM):
  - Age ≥ 1 year
  - Previous laboratory assessment and clinical evaluation consistent with iron deficiency anemia
  - Patient is not a candidate for oral iron, or has failed oral iron therapy, or has hgb < 6
- If able, obtain phosphorous level prior to administration of FCM
  - This will be followed up as outpatient and does not need to be resulted prior to giving the FCM.
  - If unable to obtain phosphorous level, appropriate to proceed with FCM infusion

- Peripheral IV placement
  - IV placement should be away from joints and/or "bends"
    - This is not a contraindication to giving the infusion if no other IV sites can be obtained.
    - Utilize arm immobilization devices
    - Instruct patients to keep arm straight and instruct patients/parents to notify RN if patient is experiencing pain during the infusion.
  - FCM may be administered through central venous access if present
- FCM administration instructions
  - FCM is ordered as 15mg/kg, with maximum one-time infusion dose of 750mg.
  - Positive (+) blood return must be documented prior to infusion of FCM
  - Vital sign frequency:
    - Prior to starting infusion
    - At the midpoint of the infusion
    - At the end of the infusion
    - Upon completion of observation period
  - Patients remain in clinical setting for a 40-minute observation period to evaluate for **hypersensitivity reactions.**

Algorithm

Infusion Reactions

Non-allergic infusion reactions:

- Flushing
- Itching
- Muscle tightness in neck, back and chest
- Headache
- Palpitations
- Joint pain

Pause infusion, when symptoms resolve can continue at ½ the initial infusion rate (discuss with pharmacy). If symptoms recur after starting, discontinue.

Monitor vital signs including blood pressure, heart rate, respirations and oxygen saturation. Close monitoring for the development of hypotension.

Do not give antihistamines as they can delay recognition of anaphylaxis.
If concern for anaphylaxis, proceed to NCH Anaphylaxis Clinical Pathway.

More serious reactions & anaphylaxis:

- Peri-orbital edema
- Cough
- Shortness of breath
- Angioedema
- Wheezing
- Stridor
- Tachycardia
- Nausea/vomiting
- Hypotension

Treat with epinephrine as indicated

[Algorithm]

[Return to Infusion Practice]

Discharge Instructions Related to Carboxymaltose Therapy

Your child received a dose of intravenous ferric carboxymaltose (Injectafer), a form of IV iron, in the Emergency Department today. Most patients tolerate the infusion well. The most common side effects appear to be increased fatigue, headache, or nausea, occurring the evening after the infusion.

There is the potential to develop low blood phosphorous levels (hypophosphatemia) after Injectafer. Most of the time, this is temporary without any symptoms, and it resolves on its own. Please contact your provider if you/your child develops mild symptoms such as symptoms such as: change in eyesight, feeling confused, mood changes, muscle pain or weakness.

Consider calling 911 if breathing problems or difficulty swallowing occurs.

Algorithm

References

1. Sarah H. O'Brien, Sherif M. Badawy, Seth J. Rotz, Mona D. Shah, Julie Makarski, Rachel S. Bercovitz, Mary-Jane S. Hogan, Lori Luchtman-Jones, Julie A. Panepinto, Ginna M. Priola, Char M. Witmer, Julie A. Wolfson, Marianne Yee, Lisa K. Hicks; The ASH-ASPHO Choosing Wisely Campaign: 5 hematologic tests and treatments to question. *Blood Adv* 2022; 6 (2): 679–685. doi: https://doi.org/10.1182/bloodadvances.2020003635

2. Shuoyan Ning, Michelle P. Zeller; Management of iron deficiency. *Hematology Am Soc Hematol Educ Program* 2019; 2019 (1): 315–322. doi: https://doi.org/10.1182/hematology.2019000034

3. Toward Optimized Practice Iron Deficiency Anemia Committee. 2018 March. Iron deficiency anemia clinical practice guideline. Edmonton, AB: Toward Optimized Practice. Available from: http://www.topalbertadoctors.org

4. Scott LJ. Ferric Carboxymaltose: A Review in Iron Deficiency. *Drugs*. 2018;78(4):479-493. doi:10.1007/s40265-018-0885-7

5. Injecatfer [package insert]. Shirley, NY: American Regent, Inc.; November 2021.

6. Rampton D, Foklersen J, et al. Hypersensitivity reactions to intravenous iron: guidance for risk minimization and management. Hematologica,2014;99(11):1671-1676.

Algorithm

Pathway Team & Process

Content Development Team:

Leaders:
Emergency Medicine:
David Kling Jr, DO

Members:
Hematology & Oncology:
Andrew Picca, DO
Amanda Jacobson-Kelly, MD
Vilmarie Rodriguez, MD
Emergency Medicine:
Berkeley L. Bennett, MD, MS
Emergency Services:
Barb Abdalla, RN, MSN, CPN
Kelli Mavromatis, RN, BSN, CPEN

Clinical Pathways Program:
Medical Director – Emergency Medicine:
Berkeley Bennett, MD, MS
Medical Director – Clinical Informatics & Emergency Medicine:
Laura Rust, MD, MPH
Business & Development Manager:
Rekha Voruganti, MBOE, LSSBB
Program Coordinators:
Tahje Brown, MBA
Tara Dinh, BS

Clinical Pathway Approved
Medical Director – Associate Chief Quality Officer, Center for Clinical Excellence:
Ryan Bode, MD, MBOE

Advisory Committee Date: *April, 2023*

Origination Date: *May, 2023*

Next Revision Date: *May, 2026*

Clinical Pathway Development

This clinical pathway was developed using the process described in the NCH Clinical Pathway Development Manual Version 6, 2022. Clinical Pathways at Nationwide Children's Hospital (NCH) are standards which provide general guidance to clinicians. Patient choice, clinician judgment, and other relevant factors in diagnosing and treating patients remain central to the selection of diagnostic tests and therapy. The ordering provider assumes all risks associates with care decisions. NCH assumes no responsibility for any adverse consequences, errors, or omissions that may arise from the use or reliance on these guidelines. NCH's clinical pathways are reviewed periodically for consistency with new evidence; however, new developments may not be represented, and NCH makes no guarantees, representations, or warranties with respect to the information provided in this clinical pathway.
Copyright © 2023. Nationwide Children's Hospital. All rights reserved. No part of this document may be reproduced, displayed, modified, or distributed in any form without the express written permission of Nationwide Children's Hospital.

For more information about our pathways and program please contact: ClinicalPathways@NationwideChildrens.org

Algorithm

Quality Measures

Goal: Appropriate evaluation and treatment of iron deficiency anemia that minimizes inappropriate blood transfusions and facilitates appropriate admissions to an inpatient unit, when indicated.

Process Metrics:

- Pathway visualization
- ED order set utilization for eligible patients discharged home

Outcome Metrics:

- Rate of patients receiving intravenous iron in the ED and are discharged home
- Rate of patients admitted and received intravenous iron
- Rate of blood transfusion amongst patients admitted for iron deficiency anemia

Balancing Measures:

- Proportion of patients seen in the ED with a return to the ED/UC within 30 days and require a blood transfusion

Algorithm
